# Metabolic and Electrophysiological Changes Associated to Clinical Improvement in Two Severely Traumatized Subjects Treated With EMDR—A Pilot Study

**DOI:** 10.3389/fpsyg.2018.00475

**Published:** 2018-04-16

**Authors:** Marco Pagani, Gianluca Castelnuovo, Andrea Daverio, Patrizia La Porta, Leonardo Monaco, Fabiola Ferrentino, Agostino Chiaravalloti, Isabel Fernandez, Giorgio Di Lorenzo

**Affiliations:** ^1^Institute of Cognitive Sciences and Technologies, Consiglio Nazionale delle Ricerche, Rome, Italy; ^2^Psychology Research Laboratory, Istituto Auxologico Italiano IRCCS, Ospedale San Giuseppe, Verbania, Italy; ^3^Department of Psychology, Universitá Cattolica del Sacro Cuore, Milan, Italy; ^4^Laboratory of Psychophysiology, Chair of Psychiatry, Department of Systems Medicine, University of Rome “Tor Vergata”, Rome, Italy; ^5^Chair of Psychiatry, Department of Systems Medicine, University of Rome “Tor Vergata”, Rome, Italy; ^6^Psychiatry and Clinical Psychology Unit, Department of Neurosciences, Fondazione Policlinico “Tor Vergata”, Rome, Italy; ^7^EMDR Italy Association, Bovisio-Masciago, Italy; ^8^Department of Nuclear Medicine, University of Rome “Tor Vergata”, Rome, Italy; ^9^IRCCS Neuromed, Pozzilli, Italy

**Keywords:** EMDR, PET imaging, EEG, neuropsychological tests, psychological tests

## Abstract

Neuroimaging represents a powerful tool to investigate the neurobiological correlates of Eye Movements Desensitization and Reprocessing (EMDR). The impact of EMDR on cortical and sub-cortical brain regions has been proven by several investigations demonstrating a clear association between symptoms disappearance and changes in cortical structure and functionality. The aim of this study was to assess by electroencephalography (EEG) and for the first time by positron emission tomography (PET) the changes occurring after EMDR therapy in two cases of psychological trauma following brain concussion and comatose state due to traffic accident. A 28 and a 29 years old men underwent extensive neuropsychological examination, which investigated: (i) categorical and phonological verbal fluency; (ii) episodic verbal memory; (iii) executive functions; (iv) visuospatial abilities; (v) attention and working memory as well as clinical assessment by means of psychopathological tests (CAPS, IES, BDI, SCL90R, and DES). They were then treated by eight sessions of EMDR. During the first session EEG monitoring was continuously performed and ^18^F-FDG PET scans, depicting brain metabolism, were acquired at rest within a week (T0). After the last session, in which the two clients were considered to be symptoms-free, neuropsychological, clinical, and PET assessment were repeated (T1). PET data were semi-quantitatively compared to a group of 18 normal controls, as for EEG the preferential cortical activations were disclosed by thresholding the individual z-score to a *p* < 0.05. There was a significant improvement in clinical condition for both clients associated with a significant decrease in CAPS scores. IES and BDI were found to be pathological at T0 and improved at T1 in only one subject. Visuo-constructive abilities and abstract reasoning improved after EMDR in both subjects. As for EEG, the most striking changes occurred in fronto-temporal-parietal cortex in subject 1 while subject 2 showed only minor changes. PET showed more pronounced metabolism in orbito-frontal and prefrontal cortex at T1 as compared to T0 in both subjects. In conclusion both clients had a clear clinical improvement in PTSD symptoms associated with metabolic and electrophysiological changes in limbic and associative cortex, respectively, highlighting the value of EMDR also in such extreme pathological conditions.

## Introduction

Post-traumatic stress disorder (PTSD) is a clinical condition that may affect victims of major psychological trauma and is one of the major contributors of mental suffering (Breslau et al., 1991; Kessler, 2000; Breslau, 2001; Darves-Bornoz et al., 2008). The traumatic event is re-experienced in flashbacks with involuntary vivid replays, concomitant autonomic reactions, and negative feelings. Leading to avoidance of reminders, irritability, and social and emotional withdrawal (American Psychiatric Association, 1994). The recurring negative trauma memory acts as new trauma experience sensitizing the brain networks engaged in fear response and resulting into the emotional bodily reactions of autonomic arousal.

In the last decades neuroimaging has represented a powerful tool to investigate the neurobiological correlates of PTSD. Consistent findings of modifications in cerebral blood flow (Single Photon Emission Computer Tomography, SPECT) (Zubieta et al., 1999; Bonne et al., 2003; Pagani et al., 2005a, 2007; Lindauer et al., 2008; Nardo et al., 2015), in metabolism (Positron Emission Tomography, PET) (Pissiota et al., 2002; Osuch et al., 2008; Molina et al., 2010; Kim et al., 2012; Zhu et al., 2016), in neuronal volume and density (Magnetic Resonance Imaging, MRI) (Lindauer et al., 2004; Looi et al., 2009; Nardo et al., 2010, 2013; O'doherty et al., 2015; Wrocklage et al., 2017), and more recently in brain electric signal (Electroencephalography, EEG) (Lee et al., 2014; Lobo et al., 2015) have been reported.

Although to date the number of studies is still quite limited, a clear implication of the limbic system, involved in processing both positive and negative emotions, in the symptomatic hyperarousal has been advocated. Upon recollection of traumatic events, the reduced medial prefrontal cortex and anterior cingulate control over hyperreactive amygdala and hippocampus initiates a pathological process thought to be the core functional mechanisms implicated in PTSD (Shin et al., 2006). However, other structures have been shown to be involved in PTSD such as thalamus (Lanius et al., 2004), insula (Chen et al., 2006; Herringa et al., 2012), Broca's area (Cottraux et al., 2015), caudate (Looi et al., 2009) as well as posterior cingulate cortex (Yamasue et al., 2003; Rogers et al., 2009).

Physical traumas might cause severe psychopathological and neuropsychological disturbances possibly resulting in PTSD symptoms and leading to metabolic and morphological changes in the brain.

Eye Movements Desensitization and Reprocessing (EMDR) is an information processing therapy for anxiety disorders focusing on trauma elaboration (Shapiro, 1989). EMDR uses upon stressful recollections alternating bilateral tactile or auditory stimulation as well as brief eye movements sets of ~30 s. Such dual task is a distinctive character distinguishing EMDR from other trauma exposure therapies. EMDR is based on the adaptive information processing model (AIP model) (Shapiro, 2001), according to which a high level of disturbance caused by traumatic experiences results in a failure of the information processing system to properly elaborate and contextualize into the semantic memory network the autobiographical event. Through EMDR the dysfunctional stored experiences will be transformed into adaptive ones, consolidating them into the natural neural processes of memory (Shapiro, 2012). Recently EMDR has been included in the most relevant international trauma treatment guidelines (United Kingdom Department of Health, 2001; Dutch National Steering Committee Guidelines Mental Health Care, 2003; INSERM, 2004; Ursano et al., 2004) and considered as evidence-based practice for the treatment of PTSD [The Substance Abuse and Mental Health Services Administration (SAMHSA), 2011], anxiety and depression symptoms (United Kingdom Department of Health, 2001).

The clinical impact of EMDR has been proven by several investigations (Högberg et al., 2007, 2008; Bisson et al., 2013; Capezzani et al., 2013; McGuire et al., 2014; Faretta et al., 2016) also demonstrating a clear association between symptoms disappearance and changes in cortical structure and functionality (Lamprecht et al., 2004; Lansing et al., 2005; Bremner, 2007; Choi et al., 2007; Pagani et al., 2007, 2012, 2013, 2015; Ohtani et al., 2009; Trentini et al., 2015; Laugharne et al., 2016).

The aim of this study was to assess by extensive neuropsychological and psychopathological test as well as by EEG and, for the first time, PET the changes occurring after EMDR therapy in two cases of psychological trauma following brain concussion due to traffic accident.

## Methods

### Subjects

Two subjects that underwent severe traffic accident, following which they were hospitalized for about 3 months in Intensive Care in a comatose state, were recruited for the study.

#### Subject 1(AR)

Twenty-nine years old man with severe head trauma caused by a motorbike accident in 2010. MRI showed several white-matter hyperintensities in fronto-parietal cortex and corpus callosum, the latter appearing thinner than usual, as well as a large post-traumatic encephalomalacia in mesio-occipital cortex. At neurological examination, a deficit of the right visual field and postural tremor of the upper limbs were found. The neuropsychological profile was characterized by impulsivity, poor inhibitory control, and impairment of working memory as well as of verbal, semantic, and visuospatial long-term memory.

#### Subject 2 (ED)

Twenty-eight years old man with severe head trauma caused by a car accident in 2009. MRI showed large hyperintense areas in cortical and subcortical right temporo-occipital and mesial frontal lobe, bilaterally. The findings were attributed to stabilized traumatic-based tissue suffering. Hypointensities of the same causal nature were described in centrum semiovale and corona radiata. Neurological examination showed a reduction of visual field, left hemiparesis with light spasticity of the upper limb and light left hemi-cerebellar syndrome with subjective instability. The neuropsychological profile highlighted deficits in reading, in visuospatial and executive functions as well as in long-term memory.

#### Controls

Eighteen participants (mean age 33 years [SD 5.86, range 22–40]; females 10/18) who were referred to the same PET center as the patients for a suspected diagnosis of cancer in whom no oncologic disease was uncovered by ^18^F-FDG-PET and who had a normal neurologic assessment served as controls. Exclusion criteria were presence of major systemic illness, major vision disturbances, psychiatric illnesses, paraneoplastic encephalitis, and diseases affecting brain functioning and metabolism.

### EMDR

The eight phases of EMDR standard protocol were carefully followed to comply with fidelity to treatment procedure and the sessions followed the standard procedures. In brief, the eight phases of the therapeutic protocol were as follows: (1). Client History: history-taking, client evaluation, identification of traumatic memories, treatment planning; (2). Preparation: stabilization and access to positive affects; (3). Assessment: guidance to accessing the perceptual, cognitive, affective, and somatic components of the disturbing memory, as well as to identifying a preferred self–referential positive cognition. Rating of feelings using the Validity of Cognition (VOC) scale, and of level of emotional disturbance by the Subjective Units of Disturbance (SUD); (4). Desensitization: focusing on the traumatic memory for about 30 s while the therapist engages in bilateral stimulation. After each set, the client reports any elicited material, which is then processed until the SUD score decreases to zero; (5). Installation: focusing on the positive cognition while recalling the memory and engaging in new sets of bilateral stimulation, until the VOC score is 7; (6). Body Scan: processing of any residual physical disturbance associated with the memories until the body is clear and free of any disturbance; (7). Closure: Completion of an EMDR session and between sessions is ensured; (8). Reevaluation: at the beginning of subsequent sessions checking whether results were kept unchanged or needed further reprocessing.

### Study design

EMDR therapy and EEGs were carried out in the private therapy room of a trained psychologist (PLP). The room was quiet and airy and therapeutic alliance was easily established. During the first session (T0), the therapist assessed the presence the psychological trauma and neuropsychological as well as neurocognitive test were administered. The two subjects were, separately, asked to record a digital file with the autobiographical narrative of their traumatic experience. After some days, they were asked to come for the second session to start EMDR therapy. EEG recording was continuously performed while the patients were:

- at rest with eyes open and closed;
- listening to the script with eyes closed;
- during a second period with eyes closed;
- during EMDR therapy;
- during a final period of rest.

The same protocol was repeated during the last EMDR session (T1), after the patient completely processed the trauma and reported no disturbance with SUD = 0, VOC = 7 and clear Body Scan.

PET scans were performed at the Department of Nuclear Medicine of the University of Rome “Tor Vergata” within a week after the first and after the last EMDR sessions.

The study was approved by the Ethical Committee of the Institute of Cognitive Sciences and Technologies and the subjects signed an informed consent and agreed to participate to the study.

### Clinical assessment

#### Neurocognitive evaluation

The two subjects underwent extensive neurocognitive testing, investigating: (i) categorical and phonological verbal fluency; (ii) executive functions; (iii) visuospatial abilities; (iv) attention and working memory (Table 1).

**Table 1:** Neuropsychological tests.

**TEST**	**Subject 1 (AR)**		**Subject 2 (ED)**	
	**T0**	**T1**	**T0**	**T1**
MMSE	15	17	20	19
Clock drawing test	**5**	1^*^	6	5
TMT A	62	58	244	293
TMT B	312	260	420	NC
TMT B-A	250	202	176	
REY imm	18	24	27	28
REY delayed	0	0	0	0
Fig REY imm	**31**	35^*^	0	5
Fig REY delayed	0	2	0	0
Digit span	5	5	**5**	6^*^
Digit span inverse	2	2	4	4
Phonemic fluency	24	25	31	25
Semantic fluency	27	29	41	24
Ideomotor apraxia	20	20	18	18
Attentive matrices	35	33	15	14
Babcock story recall	3	3,3	4	4
Babcock delayed	3	3	4	5
Frontal assessment battery	9	11	15	13
Raven progressive matrices	**29**	33^*^	**33**	35^*^

In bold^*^ the tests whose scores improved after being transformed into the Equivalent Scores.

#### Psychopathological evaluation

MINI-Plus, according to the DSM-IV criteria, assesses the presence of a wide range of psychiatric disorders including PTSD diagnosis.

CAPS measures frequency and intensity of PTSD symptoms rated for the last-week period. Seventeen items describe the classical PTSD cluster symptoms: re-experiencing, avoidance and numbing, and hyperarousal as well as symptoms associated with PTSD features. The CAPS total score ranging from 0 to 136 classifies PTSD as: 0–19: asymptomatic/few symptoms; 20–39: mild PTSD/subthreshold; 40–59: moderate PTSD/threshold; 60–79: severe PTSD symptoms; and ≥ 80: extreme PTSD symptoms.

#### Self-administered questionnaires

IES regards the response to stressful events during the past week tackling specifically areas of intrusion and avoidance. Total scores range from 0 to 75. Scores above 26 are considered to be clinically significant.

BDI measures symptoms of depression related to cognition and affection as well as to somatic changes bothering clients in the previous week (0 = not at all to 3 = severe). Total scores range from 0 to 63, with scores above 18 indicating moderate to severe depressive symptoms.

SCL-90 R reports symptoms of psychological problems in the last 7 days allowing to assess their frequency. Clients rate the items using a 5-point scale (1 = no problem to 5 = very serious). It has 3 global indexes measuring the extent or depth of individual's psychiatric disturbance; the total number of questions rated above 1 point and the intensity of symptoms.

### EEG

#### EEG procedure

The detailed EEG methodology and statistics has been described elsewhere (Pagani et al., 2011). In brief, 37-channel EEG was recorded using a pre-cabled electrode cap. Data were exported to EDF using NPX Lab 2010 (www.brainterface.com). In EMDR recordings only the epochs corresponding to the periods of bilateral stimulation were selected and exported creating files lasting several minutes. Data were analyzed in the EEGLAB environment (http://www.sccn.ucsd.edu/eeglab/index.html; Delorme and Makeig, 2004), digitally band-pass filtered between 1 and 45 Hz and re-referenced to average reference. Artifactual non-cerebral source activities were identified and rejected using a semiautomatic procedure based on Independent Component Analysis (Porcaro et al., 2009). To compute intracerebral electrical sources, we used exact low- resolution brain electromagnetic tomography (eLORETA) software (http://www.uzh.ch/keyinst/loreta.htm). Computations were made using the Montreal Neurological Institute (MNI; Montreal, Quebec, Canada) MNI152 template (Mazziotta et al., 2001), with the three-dimensional solution space restricted to cortical gray matter and hippocampi, as determined by probabilistic Talairach atlas (Lancaster et al., 2000). Intracerebral volume (eLORETA inverse solution space) was partitioned in 6,239 cubic voxels of 5 mm in which electric activity is represented for each voxel. Anatomical labels as Brodmann areas (BAs) are also reported using MNI space, with correction to Talairach space (Brett et al., 2002). Images corresponded to the estimated neuronal generators of brain activity within each band (Frei et al., 2001). The ranges of frequency bands were: delta (δ), 1.5–4 Hz; theta (θ) 4–8 Hz; alpha (α) 8–12 Hz; beta 1 (β1) 12–20 Hz; beta 2 (β2) 20–30 Hz; gamma (γ) 30–45 Hz.

Because all eLORETA inverse spatial solution voxels have a certain current density and for exploratory nature of the actual case analyses, we accepted only cluster of voxels whose Z-score was>1.5 (i.e., only the values >1.5 times the standard deviation of the standardized data in the LORETA spatial solution) and we accepted only clusters of voxels >27 voxels (an intracerebral volume cube with an edge of 15 mm).

### PET

#### Image acquisition and preliminary analysis

The two subjects fasted for at least 5 h before the i.v. of ^18^F-2-fluoro-2-deoxy-D-glucose (^18^F-FDG) infusion. Serum glucose level was a minimum of 95 mg/ml, in both of them. They were administered i.v. infusions of 210 MBq of ^18^F-FDG, were hydrated with 500 ml of NaCl 0.9% and rested 20 min in a dark silent room before undergoing PET examination.

The Discovery VCT PET/CT system (GE Medical Systems, Tennessee, USA) was used to assess FDG brain distribution in all subjects by means of a 3D-mode standard technique in a 256 × 256 matrix. Reconstruction was performed using the 3-dimensional reconstruction method of ordered-subset expectation maximization (OSEM) with 20 subsets and four iterations. The system combines a high-speed ultra 16-detector-row (912 detectors per row), a CT unit and a PET scanner with 10,080 bismuth germanate crystals in 24 rings (axial full width at half-maximum 1-cm radius, 5.2 mm in 3D mode, 157 mm axial field of view). A low-amperage CT scan of the head for attenuation correction (40 mA; 120 Kv) was performed before PET image acquisition.

### Statistical analyses

We carried out preprocessing and statistical analyses bySPM8-normalizing the images to a customized ^18^F-FDG template. The spatially normalized PET images (voxel size 2 mm) were smoothed with an 8-mm isotropic Gaussian filter. Brain PET analyses were performed, separately for each subject, before and after EMDR therapy. Individual data were compared on a voxel-by-voxel basis to those from the normal controls using a “two-sample t-test” design of SPM8 adjusted for single patient routine (Lange et al., 2016) and implemented in Matlab R2010a (MathWorks, Natick, Massachusetts, USA). The height threshold was set at a very conservative level of *p* < 0.0001 (Family Wise Correction of *p* < 0.001 at cluster level) and age and sex were used as covariate, to regress out their impact on the results. Based on the spatial resolution of the PET camera and to further improve the statistical power of the analyses only cluster larger than 92 voxels (4.5 × 4.5 × 4.5 voxels = 9 × 9 × 9 mm^3^) were considered as significant. We identified the BAs matching the SPM output to the Talairach coordinates using the subroutine implemented by Matthew Brett (http://brainmap.org/index.html).

The choice of assessing brain metabolism in the two subjects by comparing metabolism pre- and post-EMDR to a reference group was driven by the lack of statistical reliability in comparing directly the PET datasets as acquired during the first and the second session, due to the excess of noise in the single within-subject analyses.

## Results

### Subject 1(AR)

At cognitive level, AR showed post-EMDR as compared to pre-EMDR a dramatic improvement in visuo-constructive abilities and verbal memory (Clock Drawing Test and Rey immediate recall, respectively) as well as for abstract reasoning (Raven's Progressive Matrices, executive function), see Table 1.

The most striking improvement post-EMDR was in PTSD symptomatology as revealed by CAPS scores (see Table 2). Significant improvement was also found for IES, BDI, and DES scores.

**Table 2:** Psychopathological tests.

**Subject 1 (AR)**						**Subject 2 (ED)**					
	**CAPS RE-EXP**	**CAPS AVOI**	**CAPS AROU**	**CAPS ASSOC**	**CAPS TOT**		**CAPS RE-EXP**	**CAPS AVOI**	**CAPS AROU**	**CAPS ASSOC**	**CAPS TOT**
**T0**	8	22	24	6	**60**	**T0**	0	30	14	10	**54**
**T1**	2	14	6	6	28^*^	**T1**	0	21	2	2	25^*^
	**IES INTR**	**IES AVOI**	**IES TOT**				**IES INTR**	**IES AVOI**	**IES TOT**		
**T0**	23	20	**43**			**T0**	0	0	0		
**T1**	4	7	11^*^			**T1**	3	0	3		
	**BDI COG**	**BDI SOM**	**BDI TOT**				**BDI COG**	**BDI SOM**	**BDI TOT**		
**T0**	7	3	10			**T0**	1	3	4		
**T1**	3	2	5			**T1**	1	4	5		
	**SCL90R GSI**	**SCL90R PSDI**	**SCL90R PST**				**SCL90R GSI**	**SCL90R PSDI**	**SCL90R PST**		
**T0**	1.4	2.0	63			**T0**	2.2	2.7	72		
**T1**	1.3	1.9	61			**T1**	1.7	2.4	65		
	**DES**						**DES**				
**T0**	**56**					**T0**	16				
**T1**	27^*^					**T1**	28				

CAPS, Clinician-Administered PTSD Scale; CAPS–RE-EXP, CAPS re-experiencing symptoms; CAPS–AVOI, CAPS avoidant-numbing symptoms; CAPS–AROU, CAPS hyper-arousal symptoms; CAPS–ASSOC, CAPS associated features; CAPS–TOT, CAPS total score; IES, Impact of Event Scale; IES–INT, IES intrusion symptoms; IES–AVO, IES avoidance symptoms; IES–TOT, IES total score. BDI, Beck Depression Inventory. BDI–COG, BDI cognitive symptoms; BDI–SOM, BDI somatic symptoms; BDI–TOT, BDI total score. SCL-90-R, Symptom CheckList-90-Revised; SCL-90-R–GSI, SCL-90-R Global Severity Index; SCL-90-R–PSDI, SCL-90-R Positive Symptom Distress Index; SCL-90-R–PST, SCL-90-R Positive Symptom Total. In bold^*^ the remarkable decreases of tests scores.

After EMDR AR showed a substantial decrease of the re-experience (intrusive thoughts and flashback) and avoidance (meeting the people associated with the motorcycle accident) symptoms. The main improvement was in the pre-EMDR hyperarousal with a great reduction of the startle response, a regularization of the sleep-wake rhythm and a reduction of the internal tension, the latter very high before therapy.

**Figure 1:**
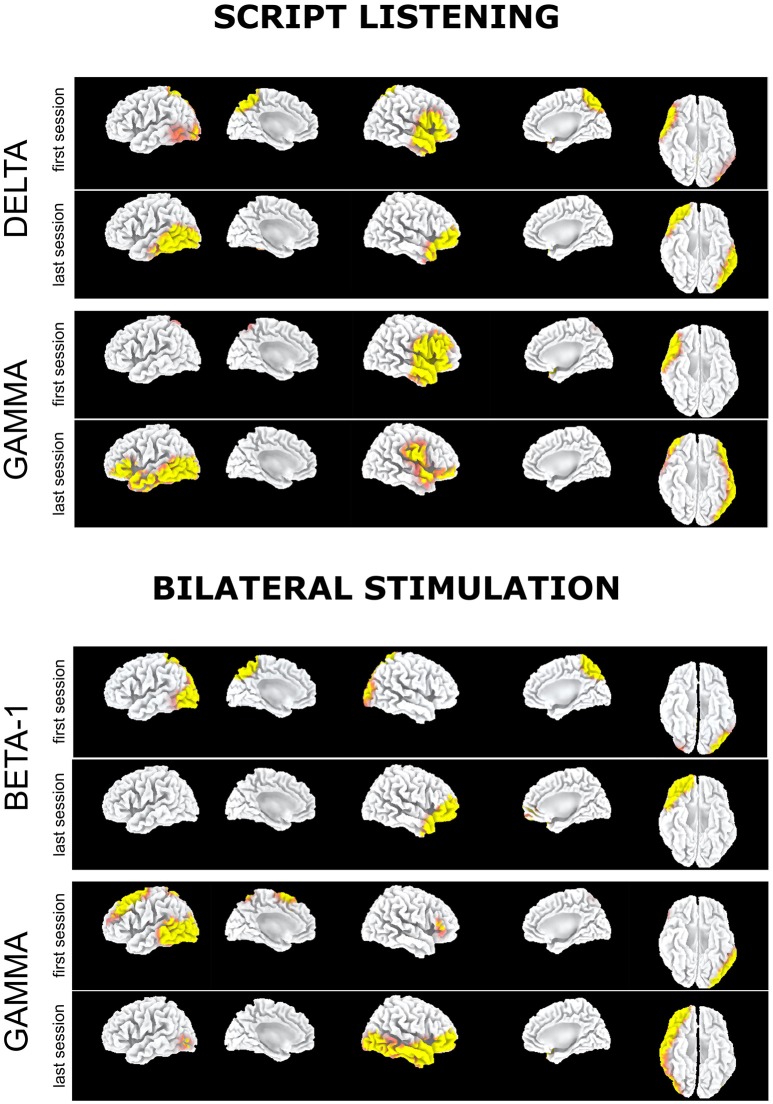
Subject 1 (AR): Delta band: Preferential activation during script listening upon the first (above) and the last (below) EMDR sessions. Gamma band: Preferential activation during script listening upon the first (above) and the last (below) EMDR sessions. Beta1 band: Preferential activation during bilateral stimulation upon the first (above) and the last (below) EMDR sessions. Gamma band: Preferential activation during bilateral stimulation upon the first (above) and the last (below) EMDR sessions.

EEG during script listening consistently showed for all bands a statistically significant disappearance during the last EMDR session as compared to the first one of the preferential cortical activation in left occipitoparietal-temporal cortex as well as in bilateral posterior cingulate/precuneus. Analogously a significant preferential activation at T1 was recorded in right prefrontal cortex and temporal pole, extending as for the gamma band to the right temporo-occipital cortex (Figure 1, script listening).

Upon bilateral stimulation and reliving of the index trauma the preferential activation in bilateral posterior cingulate/precuneus found at T0 disappeared at T1. However, at high frequencies, frontotemporal activation was found at both sessions and a large left prefrontal-temporal-occipital appeared at T1 (Figure 1, bilateral stimulation).

FDG-PET showed at T0, as compared to the control group, significant hypometabolism in left visual association cortex and right precuneus, posterior cingulate cortex and thalamus that was unchanged after therapy. On the other hand, a significant hypermetabolic area in bilateral prefrontal and anterior cingulate cortex appeared post-EMDR and the large hypermetabolic clusters found pre-EMDR in motor, temporo-parietal, and orbitofrontal cortices decreased in size and significance level after therapy (Table 3, Figure 3A).

**Table 3:** Cerebral regions showing in Subject 1 (AR) a significantly higher metabolism at PET as compared to a group of 18 control subjects.

**AR POST EMDR vs. CTRL**		**Hypermetabolic areas**					
**Cluster size**	**Cluster level**	**Peak**	**Talairach coordinates**			**Cerebral regions**	**Brodmann areas**
**equivk**	**p(FWE-corr)**	**Equivalent Z-score**	**x**	**Y**	**z**		
698	0.0000	6.15	40	−30	−15	R Fusiform Gyrus	20
		5.45	65	−43	−5	R Middle Temporal Gyrus	21
288	0.0001	5.58	46	−28	29	R Inferior Parietal Lobule	40
244	0.0003	5.54	63	−36	18	R Superior Temporal Gyrus	22
311	0.0001	5.40	−18	−31	49	L Paracentral Lobule	5
		4.99	−22	−21	45	L Precentral Gyrus	4
398	0.0000	5.09	26	16	3	R Lentiform Nucleus	Putamen
		4.73	22	15	−16	R Inferior Frontal Gyrus	47
562	0.0000	4.90	24	41	2	**R Anterior Cingulate**	**32**
		4.89	40	58	−6	**R Middle Frontal Gyrus**	**10**
181	0.0014	4.57	−18	54	−6	**L Superior Frontal Gyrus**	**10**
							
**AR PRE EMDR vs. CTRL**							
1,344	0.0000	6.11	42.0	−32.0	−17.0	R Fusiform Gyrus	20
		5.79	65.0	−49.0	−1.0	R Middle Temporal Gyrus	21
		5.70	63.0	−36.0	18.0	R Superior Temporal Gyrus	22
652	0.0000	5.62	44.0	−28.0	29.0	R Postcentral Gyrus	2
		4.54	63.0	−31.0	35.0	R Inferior Parietal Lobule	40
		5.22	−18.0	−31.0	49.0	L Paracentral Lobule	5
397	0.0000	5.08	−22.0	−21.0	45.0	L Precentral Gyrus	4
		5.13	24.0	15.0	−16.0	R Inferior Frontal Gyrus	47
458	0.0000	5.07	26.0	16.0	3.0	R Lentiform Nucleus	Putamen
		4.93	24.0	43.0	2.0	R Superior Frontal Gyrus	22
373	0.0000	4.60	28.0	48.0	−9.0	R Middle Frontal Gyrus	11

*In bold the regions showing a significant change between the two conditions*.

### Subject 2 (ED)

Post-EMDR there was an improvement in short term memory (Digit Span), semantic memory (Babcock imm) and abstract reasoning (Raven's Progressive Matrices, Table 1). Also, CAPS score decreased significantly underscoring the remarkable reduction of symptoms. On the other hand, the scores of the self-administered questionnaires were already within the normal values pre-EMDR and did not change (Table 2).

The memories of the accident were few and nonspecific both pre- and post-EMDR. After therapy ED showed an improvement in forward looking and a great reduction of the anhedonia. A reduction in avoidance (meeting people he knew before the neurological impairment occurred), irritability and neurovegetative symptoms was also observed.

**Figure 2:**
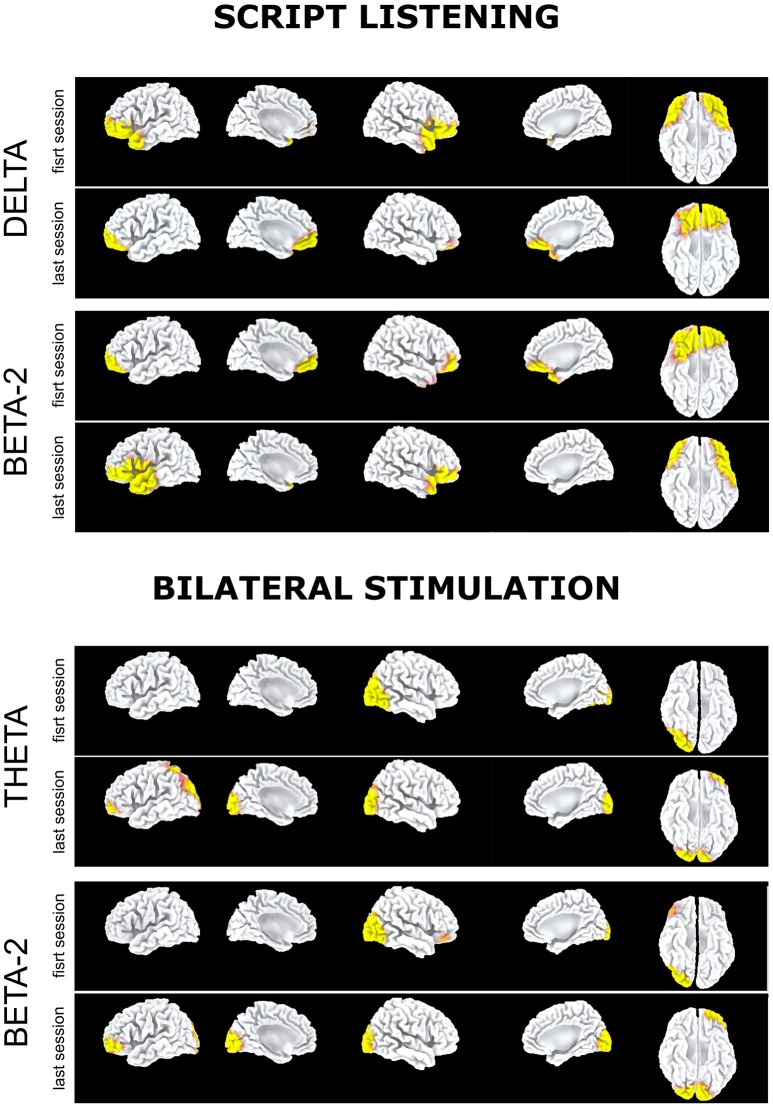
Subject 2 (ED): Delta band. Preferential activation during script listening upon the first (above) and the last (below) EMDR sessions; Beta2 band. Preferential activation during script listening upon the first (above) and the last (below) EMDR sessions; Theta band. Preferential activation during bilateral stimulation upon the first (above) and the last (below) EMDR sessions; Beta2 band. Preferential activation during bilateral stimulation upon the first (above) and the last (below) EMDR sessions.

At the last EMDR session, EEG during script listening showed a reduction at low frequencies (Figure 2, script listening, delta band) or at high frequencies (Figure 2, script listening, beta-2 band) of the preferential cortical activation found during the first session in bilateral prefrontal cortex and temporal pole.

**Figure 3:**
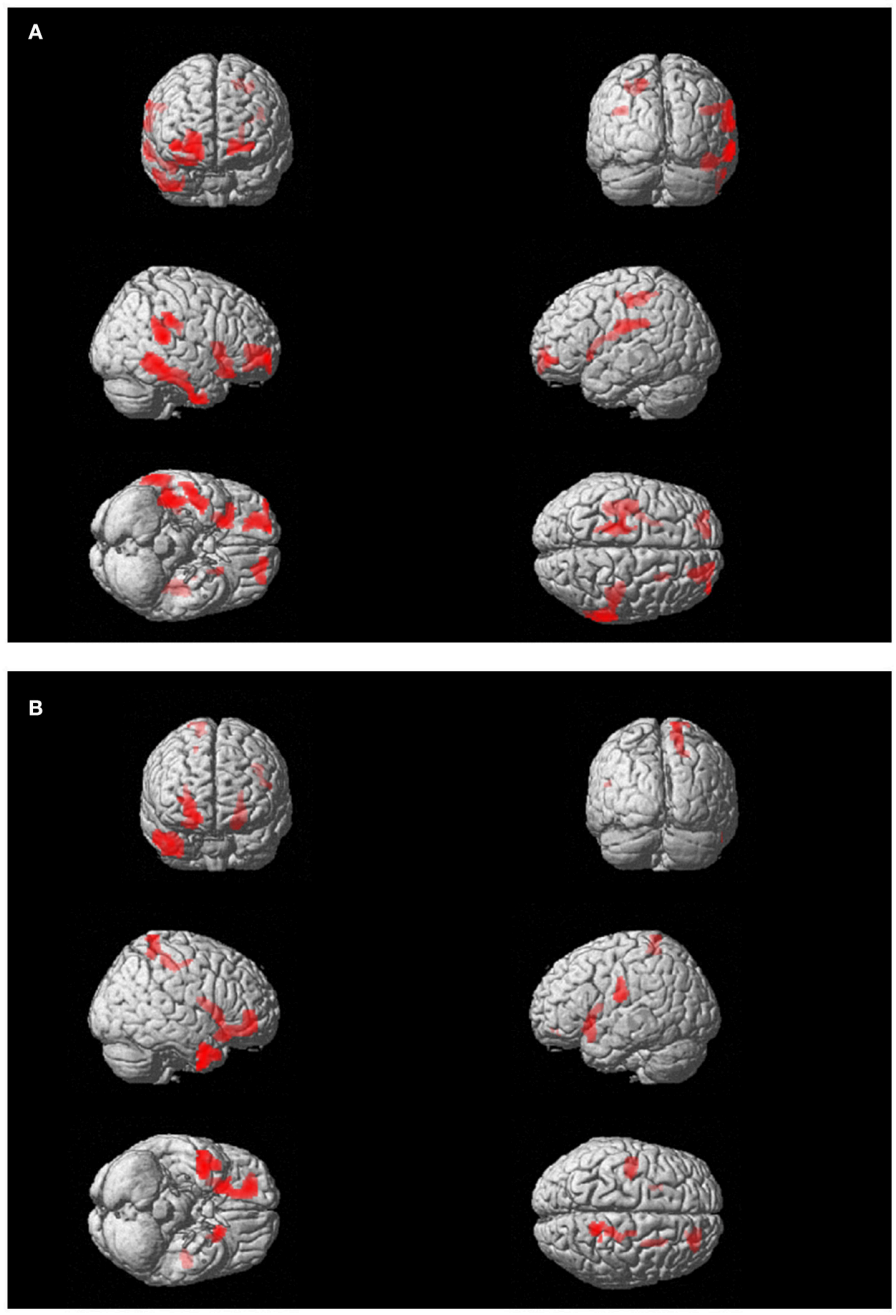
(A) Subject 1 (AR): PET findings in AR post-EMDR compared with 18 control subjects. Statistically significant different hypermetabolic regions p(FWE-corr) are highlighted on a glass-brain template. Top left frontal view; top right posterior view, middle right-side view; middle right: left-side view; bottom left: view from below; bottom right: view from above. **(B)** Subject 2 (ED): PET findings in ED post-EMDR compared with 18 control subjects. Statistically significant different hypermetabolic regions p(FWE-corr) are highlighted on a glass-brain template. Top left frontal view; top right posterior view, middle right-side view; middle right: left-side view; bottom left: view from below; bottom right: view from above.

Such preferential activations were not recorded during bilateral stimulation in which at T1 as compared to T0 an increased cortical activation was found in left parieto-occipital cortex in theta band (Figure 2, bilateral stimulation). Notably in this subject during bilateral stimulation an activation in right associative visual cortex was systematically found during both sessions and a faint activation in left prefrontal cortex appeared at T1 in theta and beta-2 bands (Figure 2, bilateral stimulation).

FDG-PET highlighted the disappearance during the last session of the hypometabolism found at T0, beside several temporal and anterior and posterior cingulate areas (Table 4), in parahippocampal and fusiform gyrus. In agreement with the EEG findings, at T1 a relatively higher metabolism was found in right precuneus, parietal, and posterior cingulate cortex as compared to T0 as well as a relative hypermetabolism in left putamen and orbitofrontal cortex (Table 5, Figure 3B). The hypermetabolism found at T0 in right orbitofrontal and temporal cortex did not substantially changed.

**Table 4:** Cerebral regions showing in Subject 2 (ED) a significantly lower metabolism as compared to a group of 18 control subjects.

**CTRL vs. ED POST EMDR**		**Hypometabolic areas**					
**Cluster size**	**Cluster level**	**Peak**	**Talairach coordinates**			**Cerebral regions**	**Brodmann areas**
**equivk**	**p(FWE-corr)**	**Equivalent Z-score**	**x**	**y**	**z**		
4,174	0.0000	6.26	61	−40	−15	Right Inferior Temporal Gyrus	20
		5.78	12	−29	0	Right Thalamus	^*^
364	0.0000	5.06	−4	−30	29	Left Posterior Cingulate	23
		4.81	6	−28	29	Right Posterior Cingulate	23
2,596	0.0000	4.91	4	19	36	Right Anterior Cingulate	32
		4.80	−2	43	13	Left Anterior Cingulate	32
**CTRL vs. ED PRE EMDR**							
4,270	0.0000	6.27	61	−44	−15	Right Middle Temporal Gyrus	20
		5.74	12	−29	0	Right Thalamus	^*^
400	0.0000	4.98	−4	−30	29	Left Posterior Cingulate	23
		4.75	6	−26	31	Right Posterior Cingulate	23
1,919	0.0000	4.96	2	25	28	Right Anterior Cingulate	32
		4.71	−2	45	14	**Left Medial Frontal Gyrus**	**9**
		4.94	−22	−15	−28	**Left Parahippocampal Gyrus**	**35**
122	0.0076	4.90	0	−61	20	Left Precuneus	23
133	0.0054	3.94	8	−56	14	Right Posterior Cingulate	23
122	0.0076	4.08	−22	−51	−9	**Left Fusiform Gyrus**	**37**

*In bold the regions showing a significant change between the two conditions*.

**Table 5:** Cerebral regions showing in Subject 2 (ED) a significantly higher metabolism at PET as compared to a group of 18 control subjects.

**ED POST EMDR vs. CTRL**		**Hypermetabolic areas**					
**Cluster size**	**Cluster level**	**Peak**	**Talairach coordinates**			**Cerebral regions**	**Brodmann areas**
**equivk**	**p(FWE-corr)**	**Equivalent Z-score**	**x**	**y**	**z**		
786	0.0000	5.44	22	42	−9	Right Middle Frontal Gyrus	11
		5.13	22	15	−16	Right Inferior Frontal Gyrus	47
		4.86	26	16	1	Right Lentiform Nucleus	Putamen
515	0.0000	5.22	40	0	−34	Right Inferior Temporal Gyrus	20
		4.67	53	1	−24	Right Middle Temporal Gyrus	21
		4.58	44	12	−29	Right Superior Temporal Gyrus	38
230	0.0004	5.11	−46	−13	19	**Left Postcentral Gyrus**	**43**
285	0.0001	4.77	18	−29	49	**Right Paracentral Lobule**	**5**
		4.61	18	−44	57	**Right Precuneus**	**7**
		4.35	22	−23	45	**Right Posterior Cingulate**	**31**
234	0.0003	4.64	−22	15	−4	**Left Lentiform Nucleus**	**Putamen**
		4.37	−22	13	−16	**Left Inferior Frontal Gyrus**	**47**
							
**ED PRE EMDR vs. CTRL**							
759	0.0000	5.27	24	42	−7	Right Middle Frontal Gyrus	11
		5.16	18	31	−8	Right Sub-Gyral	47
		5.14	26	16	3	Right Lentiform Nucleus	Putamen
322	0.0000	4.90	40	0	−30	Right Middle Temporal Gyrus	21
		6.44	44	10	−29	Right Superior Temporal Gyrus	38

*In bold the regions showing a significant change between the two conditions*.

## Discussion

The aim of the present study was two-fold: (i) evaluate the efficacy of EMDR in treating the post-traumatic psychological sequelae of life-threatening traffic accidents followed by a comatose state; (ii) perform a complete neurobiological evaluation of the therapy outcome adding the metabolic status to the assessment of the cortical electrical activity.

The general neurocognitive status was not substantially modified by EMDR therapy with poor performances in the most of neurocognitive test both before and after therapy. However, in both subjects there was an improvement in the scores of tests reflecting abstract reasoning and verbal memory and AR had a significant progress toward the normative values in visuospatial abilities (Table 1). The improvement in these constructs was possibly associated at T1 to a better attention and verbal understanding as well as to a reduction in depressive mood that might have influenced the results of the tests pre-EMDR.

EMDR was very effective in reducing PTSD symptoms in both subjects. CAPS scores decreased significantly in all subscales (relieving, avoidance, arousal, and association) and the two subjects showed clinical improvement with symptoms reduction (Table 2). In both subjects, there was a decrease in avoiding either people associated to the accident (AR) or known before the neurological impairment occurred (ED). Furthermore, all symptoms reported pre-EMDR as intrusive thoughts, hyperarousal, sleep disturbances, irritability, and vegetative symptoms were greatly reduced.

Furthermore, for AR the significant decrease of the scores of IES, BDI, and DES scales spoke in favor of a post-EMDR drop of post-traumatic, depressive, and dissociative symptoms. The appropriateness of self-administered questionnaires to depict neurobiological changes occurring before and after therapy has been demonstrated by two recent investigations in which the neuropsychological scores highly correlated with the activation induced by trauma exposure in the same regions in which functional changes between the two conditions were found (Nardo et al., 2011; Trentini et al., 2015).

Post-Traumatic stress disorder results in well-known alterations affecting cortical and subcortical regions. Several studies converge in ascribing to the hyperactivation of the limbic structures and to an insufficient cortical control upon reliving of negative emotion the neurobiological core of PTSD (Shin et al., 2006). Decreased top-down cognitive control of the prefrontal and the anterior cingulate cortices results during stressful conditions in an abnormal response of amygdala, hippocampus, and insula causing PTSD symptoms to appear. On the other hand, inconsistent results have described (reduction or no changes) in hippocampal volume and a few investigations have reported gray matter volume or density reduction in other structures (Nardo et al., 2010, 2013; O'doherty et al., 2015, 2017; Wrocklage et al., 2017).

EMDR has been recognized as elective treatment in reducing PTSD symptoms (Lehman et al., 2004; Bisson et al., 2013; Tol et al., 2013) and it has also been proven to be useful in other pathological conditions as depression (Acarturk et al., 2018); bipolar disorder (Moreno-Alcazar et al., 2017), chronic pain (Tesarz et al., 2014), and substance use disorder (Schafer et al., 2017). To the best of our knowledge this is the first study investigating EMDR efficacy also in case of severe physical trauma followed by brain anatomical and functional changes. Furthermore, in both the investigated cases clinical evidence of psychopathological and cognitive symptoms was still actual after more than 8 years from the event, suggesting the presence of a chronic PTSD associated with deteriorated brain conditions. Both subjects suffered of extensive neuronal damage. White matter as well as gray matter structural changes were found in cortical and subcortical regions with large post-traumatic encephalomalacia in occipital cortex, possibly consequence of the severe brain concussion. Furthermore, both subjects showed neurological deficits with impairment in motor, visuospatial, and various cognitive functions.

In our study, the neurobiological effect of EMDR went beyond the normalization of the dysfunctional cortico-limbic response as demonstrated by recent studies (Pagani et al., 2012, 2015; Trentini et al., 2015) and could overcome the impact that organic damage had on subjects' psychopathology.

For the first time, PET was performed to test the metabolic changes following EMDR therapy. Previous studies revealed following Cognitive Behavioral Therapy decreased FDG-PET resting state glucose metabolism in frontal regions and increased metabolism in anterior cingulate gyrus and related regions (Goldapple et al., 2004) as well as increased metabolism in anterior cingulate gyrus and related regions (Kennedy et al., 2007). Accordingly, SPECT investigations assessing cerebral blood flow (CBF), normally coupled with metabolism and performed before and after psychotherapy, showed frontal CBF changes associated with symptoms disappearance (Lansing et al., 2005; Pagani et al., 2007; Peres et al., 2007; Lindauer et al., 2008). Functional neuroimaging methodologies have been refined in the last decades with improvements in whole brain (Friston et al., 1991) and regional (Thurfjell et al., 2000) analyses. Both SPECT (Bonne et al., 2003; Pagani et al., 2005b, 2007; Lindauer et al., 2008; Nardo et al., 2015) and PET (Bremner et al., 1999, 2003; Shin et al., 1999, 2004; Gilboa et al., 2004) investigations have been performed to disclose in PTSD the regional activations upon the reliving of the traumatic event submitting to the experimental subjects sensory stimuli (visual or auditory), often with a strong autobiographical connotation. Such studies have been conclusive in disclosing the neurobiological model of PTSD above described.

Due to the dynamics of the accidents and of the subsequent brain damage we found in the two subjects at T0 in both EEG and PET examinations different electrical cortical and metabolic patterns which changed significantly after EMDR therapy.

It has to be underscored that in the present study EEG was recorded during active emotional stimulations (script listening or during phase four of EMDR therapy) while PET examinations were performed in the so-called resting state, when a participant is asked to lie quietly in the scanner without performing any specific task. In this condition signal increases and decreases are due to spontaneous or “intrinsic” neuronal fluctuations upon radiopharmaceutical administration and the following 10–20 min in which the concentration in the brain reaches a steady-state. Metabolic resting-state data depict the pattern characteristic of normal psychology and psychological disorders as well as psychological functions associated with the self. In the case of traumatized individuals, the rumination and the mental wandering within the scanner gantry may result, beyond the neurodegenerative status, in hypo-or hyperactivations characterizing their psychopathological status and hence being unique for each subject. In our two subjects, PET was able to capture such condition of distress and represent its metabolic pattern. As expected from the MRI findings, AR showed a severe hypometabolism in left visual association cortex in the occipito-parietal lobes that did not show any change post-EMDR. As compared to the control subjects, large areas of hypermetabolism that were found in temporoparietal cortex at T0 diminishing significantly at T1 (Table 3). However, in accordance with the EEG findings during both script listening and bilateral stimulation a new hypermetabolic cluster appeared in prefrontal and anterior cingulate cortex, speaking in favor of a possible better top-down control on the subcortical hyperarousal (Figures 1, 3A). A similar concordance of findings was present in ED in which EEG upon bilateral stimulation showed at T1 an activation of the right prefrontal cortex similar to the one found post-EMDR by PET. As discussed above the two methodologies as applied in the present study are different in nature. EEG captured the preferential cortical electrical activation during specific tasks while PET was performed in a (theoretical) resting state, depicting a more static metabolic status. However, it can be speculated that following successful EMDR therapy state (EEG) and trait (PET) conditions may converge into similar patterns. In this respect, the increased relative activation of prefrontal cortex at T1 in both subjects and disclosed by both methodologies may reflect as the successful attempt of cortical structures to reduce the subcortical hyperarousal, hypothesis supported by the great improvement in clinical status.

On the other hand, beside the changes toward a more preeminent activity in prefrontal cortex, both methodologies showed changes in other regions. EEG showed in AR at T1large shifts in cortical activation in temporo-occipital cortex (Figure 1), previously identified as the region mostly activated when PTSD symptoms disappear following successful EMDR therapy (Pagani et al., 2012, 2015) while in ED the activation state induced by both script listening and traumatic exposure was pretty similar in both conditions (Figure 2).

Similarly, metabolism in both subjects showed not only the prefrontal and anterior cingulate increase at T1 but increased in both conditions as compared to the control group in temporo-parietal regions (Tables 3, 5) probably due to ruminating emotional thoughts during radiopharmaceutical administration and time before the scanning. In ED, the reduction of PTSD symptoms was associated with the disappearance of the hypometabolism in parahippocampal cortex and in the fusiform gyrus, regions known to be implicated in the pathophysiology of PTSD (Table 4).

In the case of PET, the metabolic changes were not assessed by a within-subject experimental design since the background noise (signal variability) in individual scan would have been excessive for a one-to-one scan comparison making the results of the analyses unreliable. We then chose to compare the data of the pre-EMDR PET scan of each subject to a set of scans of age-matched controls and run the same comparison again using the post-EMDR PET data. This kind of analysis was recently validated (Lange et al., 2016) and it is currently used in clinical setting to assist physicians in the diagnosis of neurodegenerative diseases (Gallivanone et al., 2017). In this way, we matched to the very same reference (eighteen control subjects) the individual scans pre- and post-EMDR being able to appreciate the changes occurring from the first to the second condition.

As the most of pilot studies, the present investigation suffers of some limitations, due to the inherent nature of the experiment (case reports). We did not include into the design of the study subjects undergoing the same traumatic event and not treated by EMDR. This prevented a controlled cause-effect link between the improvement of clinical condition and psychotherapy as well as the generalizability of our results to other cases of traumatic brain injury. Recruiting such individuals would have required an extensive retrospective screening identifying subjects with similar characteristic and duration of symptoms. It would have needed resources not available and was beyond the purpose of the present pilot investigation. However, the study was tailored for the two subjects according to their clinical condition and needs. Indeed, they were suffering since many years of post-traumatic symptoms related to the brain concussion as a result of traffic accident. We believe that the fact that EMDR could clearly mitigate the post-traumatic symptoms after more than 8 years in which they did not undergo any psychotherapy is a proof of concept of its effectiveness.

This limitation might be overcome by the recruitment in a prospective study of individuals suffering traumatic brain injury and randomized after the acute phase into two groups, one treated as usual and the other by EMDR. Such experimental design along with careful neuropsychological, neurophysiological and metabolic assessment of the respective outcomes might more reliably support the conclusions of the present pilot study. The hypothesis of this design would have to differ slightly from the present study. Because chronic PTSD would not have had time to develop, outcome measures would include incidence and severity of long-term psychological trauma in addition to comparative metabolic and electrophysiological changes in the two groups.

The two subjects showed a neurobiological response that was not directly comparable and, mainly at EEG, changes occurred in different regions. These inconsistencies derive firstly from the different individual response to emotional trauma exposition and to therapy, associated to the differences in pattern of injury between the two individuals underlying their neurocognitive state and secondly from the different neurological and anatomical functional deficits that each subject suffered as a result. Furthermore, the latter structural changes caused both PET and EEG to detect regional changes very likely deriving also from disrupted neuronal networks resulting in patterns of metabolism and cortical activation not applicable to a population of patients suffering of traumatic brain injury in which the anatomical damage and functional impairment vary from case to case.

Following the present promising pilot study in the next future attempts might be performed to investigate ^18^F-FDFG-PET upon exposure to a psychological stress following a recent study in which olfactory stimulation was administered for about 10 min to the experimental subject (Chiaravalloti et al., 2015). Such experimental protocol would enable a direct correspondence upon traumatic exposure between electrical cortical activity and metabolic response allowing a better definition of the limbic and cortical regions implicated in the emotional process, due to the better spatial resolution of PET for sub-cortical structures as compared to EEG.

In conclusion, EMDR was proven to be clinically useful in two difficult cases of chronic PTSD due to severe physical trauma. This first ever investigation combining neuropsychological and psychopathological tests, EEG, PET, and EMDR yielded very promising results showing neurobiological changes following successful therapy as revealed by all measurements. The refinement of PET procedures allowing a dynamic assessment of the metabolic changes and the use of EEG instruments with larger number of sensors and more sophisticated software will in the future allow to more deeply investigate the association between electric cortical activity and metabolic changes.

## Author contributions

MP, GC, IF and GD: study concept and design; PL, AD, LM, GD, AC, and FF: acquisition of data; MP, AC, AD, LM, GD, and FF: analysis and interpretation of data; MP, GC, AD, and FF: Drafting of the manuscript; MP, GC, AD, IF and AC: Critical revision of the manuscript for important intellectual content; MP and IF: obtained funding; AD, LM, FF, and AC: administrative, technical, and material support.

### Conflict of interest statement

The authors declare that the research was conducted in the absence of any commercial or financial relationships that could be construed as a potential conflict of interest.
